# An Overview of *Trypanosoma brucei* Infections: An Intense Host–Parasite Interaction

**DOI:** 10.3389/fmicb.2016.02126

**Published:** 2016-12-26

**Authors:** Alicia Ponte-Sucre

**Affiliations:** Laboratory of Molecular Physiology, Institute of Experimental Medicine, Luis Razetti School of Medicine, Faculty of Medicine, Universidad Central de VenezuelaCaracas, Venezuela

**Keywords:** human African trypanosomiasis, variant surface glycoprotein, dynamic interaction, antigenic diversity, host response

## Abstract

*Trypanosoma brucei rhodesiense* and *T. brucei gambiense*, the causative agents of Human African Trypanosomiasis, are transmitted by tsetse flies. Within the vector, the parasite undergoes through transformations that prepares it to infect the human host. Sequentially these developmental stages are the replicative procyclic (in which the parasite surface is covered by procyclins) and trypo-epimastigote forms, as well as the non-replicative, infective, metacyclic form that develops in the vector salivary glands. As a pre-adaptation to their life in humans, metacyclic parasites begin to express and be densely covered by the Variant Surface Glycoprotein (VSG). Once the metacyclic form invades the human host the parasite develops into the bloodstream form. Herein the VSG triggers a humoral immune response. To avoid this humoral response, and essential for survival while in the bloodstream, the parasite changes its cover periodically and sheds into the surroundings the expressed VSG, thus evading the consequences of the immune system activation. Additionally, tools comparable to quorum sensing are used by the parasite for the successful parasite transmission from human to insect. On the other hand, the human host promotes clearance of the parasite triggering innate and adaptive immune responses and stimulating cytokine and chemokine secretion. All in all, the host–parasite interaction is extremely active and leads to responses that need multiple control sites to develop appropriately.

## Introduction

Few microorganisms are responsible for the major parasitic diseases of the XX and XXI centuries, trypanosomes among them. HAT, or sleeping sickness, is a severe meningoencephalitic disease that develops after an acute lymphatic infection. The estimates of “years lived with disability” for HAT, according to the Global Burden of Disease, range from 2,000 to 25,000 per year, with about 30 African countries affected by the ailment (Sutherland et al., 2015). However, the rate of under-reporting may reach numbers higher than 40%, meaning that the accurate figures of people at risk are underestimated (Fèvre et al., 2008).

Similar to other microbes and parasites, trypanosomes challenge the immune system and induce a host response. This parasite–host interaction can produce either a poor immune response, with a consequent devastating hyper-infection, or an exaggerated life threatening immune response, also with overwhelming consequences. To be successful, the parasite needs to poise its behavior between these two extremes, avoiding indiscriminate killing of the host and still escaping destruction by the immune system. Trypanosome achievement has been to develop successful means of evading the consequences of the immune system activation and this might be an outcome of the time constraints and periods shared with humans over their evolution for many million years (Steverding, 2008; Cnops et al., 2015).

Except for *Trypanosoma cruzi*, that invades host cells and is thus an intracellular pathogen, trypanosomes are extracellular parasites. When infective trypanosomes invade the bloodstream, a humoral immune response is usually triggered. However, *T. brucei* parasites successfully eludes the adaptive immune response through the expression of the antigenic VSG. Although it might be contradictory to use the main antigenic molecule exposed in the surface of the parasite as a protective tool, the VSG expressed in the parasite membrane is constantly shed and renewed by means of re-arrangements in the genome. Covering the cell with a frequently changed “protective coat” represented by the VSG allows the parasite to avoid detection by host antibodies (Horn, 2014).

Switching to a new VSG coat thus constitutes a central mechanism for the effective evasion of the mammalian immune system (Horn and Mcculloch, 2010). Additionally, a mechanism analogous to quorum sensing contributes to the successful parasite transmission from mammal to insect (Seyfang et al., 1990; Morrison, 2011). The host activates innate immunity and cytokine and chemokine secretion to trigger the clearance of the parasite. This all means that the interaction between the mammalian host and the parasite is extremely active. Herein, we will briefly discuss the focused strategy used by the parasite and question whether or not there is a truly developed host response, since by using the VSG-switch to facilitate the escape of a subpopulation of trypanosomes from antibody-mediated killing the parasite circumvents the host immune system.

## Diversity of *Trypanosoma* Species and Hosts

Most species of trypanosome are incompetent to infect man; they infect animals, mainly through their natural insect vector, the tsetse fly. The trypanolytic protein apolipoprotein-L1 and two protein complexes or TLF present in human serum (TLF1 and TLF2) provide innate resistance and normally prevent human infections (Pérez-Morga et al., 2005; Molina-Portela et al., 2008; Capewell et al., 2015).

Nevertheless, *T. brucei gambiense* and *T. brucei rhodesiense*, that belong to the *T. brucei* (*b.*) group (subgenus *Trypanozoon*), are the species that cause HAT. Both species express mechanisms that overcome and neutralize the function of TLF1 and TLF2 factors. In *T. b. rhodesiense* the serum resistance associated gene, which is a truncated VSG, confers resistance to lysis. In *T. b. gambiense* a more sophisticated system performs this task. It involves a specific truncated VSG (TgsGP), a reduced binding affinity of its receptor for TLF and an increased cysteine protease activity (De Greef et al., 1992; Xong et al., 1998; Kieft et al., 2010; Stephens and Stephen, 2011; Capewell et al., 2015). Of note, several populations of non-human primates display resistance to infection when challenged with *T. b. gambiense* and *T. b. rhodesiense* (Capewell et al., 2015).

*Trypanosoma brucei rhodesiense* causes the more virulent form of HAT (East African or Rhodesian African sleeping sickness). The Rhodesian African sleeping sickness is zoonotic and rare; patient deaths often occur within a few months. The West African or Gambian African HAT disease caused by *T. b. gambiense* displays long latency and chronicity (Franco et al., 2014). For this latter form of disease human beings are the main reservoir and transmission agent within the life cycle of the parasite. Unfortunately, there is neither a vaccine nor a recommended drug available to prevent any (West or East) African trypanosomiasis (La Greca and Magez, 2011).

Blood-sucking infected female tsetse flies (genus *Glossina spp.*; Steverding, 2008) transmit HAT. Within the vector, procyclic parasites proliferate and the parasite goes through transformations until reaching the metacyclic stage, i.e., the infective form for the human host. The metacyclic form that develops in the vector salivary glands may be inoculated by the tsetse fly with its saliva into a mammalian host (Franco et al., 2014). HAT is infrequently transmitted by blood transfusion (Franco et al., 2014). The disease causes infertility and miscarriage in humans; thus, transmission by placental means is uncertain. Still, the guidelines and protocols related to HAT stress that pregnant women and newborns from infected mothers should be systematically checked for HAT (Lindner and Priotto, 2010; Franco et al., 2014). Oral, blood–blood (e.g., by sexual contact), and iatrogenic transmission (with contaminated needles) could have an occasional impact (Franco et al., 2014). Mechanical transmission by biting insects that suck pathogens from one host (Gruvel, 1980) and prior to swallowing its blood inoculate them with the saliva into another host (Foil, 1989), might be relevant for transmission of pathogenic trypanosomes that do not infect humans, but its epidemiological impact for the human disease is difficult to assess.

Two African trypanosome subgenera, *Nannomona* and *Duttonella* (*T. congolense* and *T. vivax*, respectively), cause the cattle disease nagana (Duffy et al., 2009; Morrison et al., 2009). *T. evansi* (subgenus *Trypanozoon*) infects mammals including horses, mules, camels, buffalo, cattle and deer and produce a disease called surra (*mal de cadeiras*), of great economic importance in many geographic areas. In fact, in Africa, Asia, and South America thousands of animals die from *T. evansi* or *T. vivax* infection every year (Camargo et al., 2015). *T. equiperdum* (subgenus *Trypanozoon*) infects equines under natural conditions; by venereal transmission it may also cause a disease of equines called dourine (Desquesnes et al., 2013). *T. evansi* (monomorphic) and *T. equiperdum* (monomorphic but occasionally pleomorphic) are morphologically similar to the slender forms of *T. b. brucei*, as well as of *T. b. rhodesiense* and *T. b. gambiense* (Blacklock and Yorke, 1993; Brun et al., 1998). The information about the host response in infections produced by these veterinarian parasites is limited (Lemos et al., 2008; Camargo et al., 2015); therefore, the present review deals mostly with the human infecting trypanosomes that cause HAT.

## Clinical Presentation of Human African Trypanosomiasis

In HAT caused by members of the *T. brucei* group, i.e., *T. b. rhodesiense* and *T. b. gambiense*, the comprehension of the role played both by the parasite and the host during the host–parasite interaction is essential to understand the clinical performance of the disease.

The sickness has two clear cut clinical stages, i.e., a hemolymphatic initial systemic stage and a second phase characterized by the invasion of the brain by parasites. This encephalitic stage involves sensory, motor and psychiatric disturbances, with alterations of sleep representing the most typical manifestations.

**FIGURE 1:**
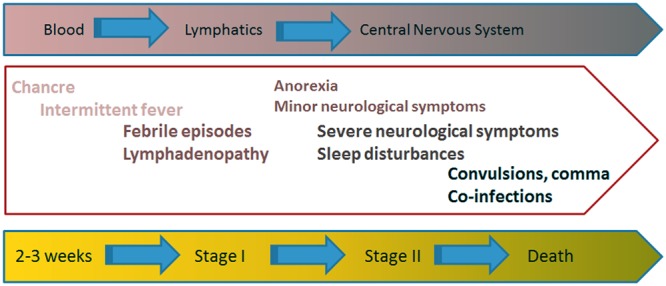
**Clinical presentation of HAT.** The painful (infected) tsetse bite leads to the appearance of the so-called chancre. Two or three weeks later the disease evolves into two successive clinical stages. The hemolymphatic stage I is characterized by the appearance of intermittent fever episodes, adenopathies, splenomegaly, and even hepatic disturbances. Painful lymph nodes also occur at this stage. The meningoencephalitic stage II appears slowly over a period of months or years, depending on the infecting trypanosome. The parasites cross the blood brain barrier to infect the CNS, causing the appearance of the neurological manifestations characteristic of HAT, like disturbance to the patient’s sleep patterns, confusion and difficulty with coordination. At the terminal phase of the disease, disturbances in consciousness and the development of dementia with incoherence, double incontinence and epileptic seizures are common.

The clinical presentation of HAT is thoroughly described by Vincendeau and Bouteille (2006) and I will briefly summarize its main steps. **Figure 1** reviews the stages that portray the clinical symptoms of HAT. The tsetse bite produces a painful reaction characterized by local erythema, heat, edema, and tenderness that leads to the appearance of the chancre. This is an ulcer where the parasites are present and it disappears after 2 or 3 weeks. Thereafter the disease evolves into the successive phases that characterize its clinical phases (Sternberg, 2004; Kennedy, 2013).

### The Hemolymphatic Stage I

Shortly after being infected, the patient enters the hemolymphatic stage I, a phase in which the disease is often undiagnosed and, therefore, untreated. Intermittent fever episodes often occur as a consequence of the successive cyclical waves of trypanosome parasitemia. However, febrile occurrences cannot be considered as useful diagnostic elements since they might be completely absent. Adenopathy, splenomegaly and even liver disturbances signal the invasion of the reticuloendothelial system by trypanosomes. Skin rashes and severe pruritus with scratching skin lesions might become unbearable for the patient and painful lymph nodes might also occur at this stage (Sternberg, 2004; Kennedy, 2013).

### The Meningoencephalitic Stage II

The meningoencephalitic stage II is an insidious phase that appears slowly over a period of months or years. The parasites cross the blood–brain barrier and infect the CNS, causing disturbances to the patient’s sleep pattern, as well as confusion and trouble with motor and mental coordination. Fever spikes common to the hemolymphatic stage I might still be present. Abnormal quantities of macrophages enter the CSF and non-specific perivascular inflammatory cell infiltrates occur in the leptomeninges and white matter, with a pronounced activation of microglia and astrocytes (Sternberg, 2004; Kennedy, 2013).

The broad spread of the meningeal inflammation to specific CNS locations leads to the appearance of the neurological symptoms common to HAT. For example, invasion of the median eminence by parasites may be the cause of sleep-wake abnormalities. At the terminal phase of the disease, disturbances in consciousness and the development of dementia with incoherence, double incontinence and seizures are consequences of CNS demyelination and atrophy (Cnops et al., 2015). The patient dies by heart failure or by encephalitis in a state of cachexia and physiological collapse (Sternberg, 2004; Kennedy, 2013).

## Transmission Biology of Human African Trypanosomiasis

To cope with the complexity of events involved in the host–parasite interaction trypanosomes use a focused strategy that includes the development of distinct life stages to adapt themselves to the environment they encounter (Vickerman, 1965, 1985; Matthews and Gull, 1994).

**FIGURE 2:**
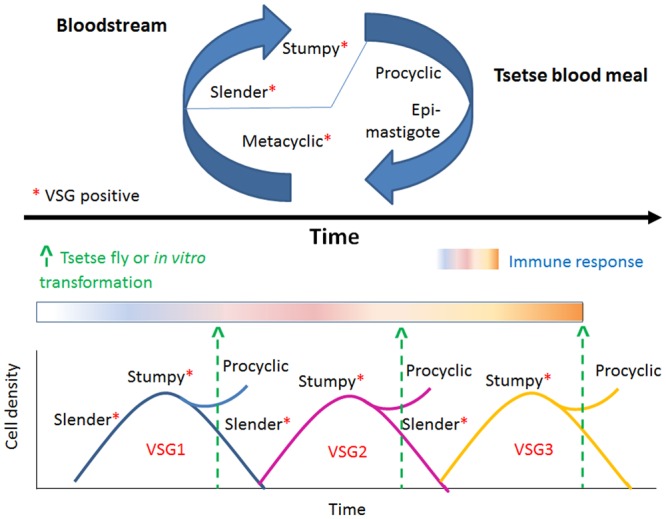
**The changing aspects of VSG expression.** Trypanosomes oscillate between distinct life stages. Metacyclic (VSG [+]) parasites become proliferative slender (VSG [+]) forms in the blood stream, reach G1/G0 phase and differentiate to non-proliferative, transmissible, stumpy, G0 arrested, VSG [+] forms. Stumpy forms only transform efficiently within the tsetse midgut to procyclic epimastigote forms (VSG [-]). VSG is constantly changed in each transmission cycle. This strategy represents an advantage for the parasite that the human immune system cannot control and thus warrants the parasite successful evasion of the immune system surveillance.

During the active interaction between the host and the parasite the active balance between slender and stumpy trypanosome forms play key roles in infection and transmission as is depicted in **Figure 2**. That is, in the bloodstream, the parasite proliferates (slender parasite forms with asynchronous life cycle) and eventually reaches the G1/G0 cell cycle phase and becomes arrested at the non-proliferative, transmissible, stumpy (G0 arrested, synchronous differentiated) forms (Vickerman, 1965, 1985), to be transmitted to the vector. Herein I will briefly describe the events that characterize the human host–parasite and vector-parasite interfaces.

### Bloodstream Parasites

**FIGURE 3:**
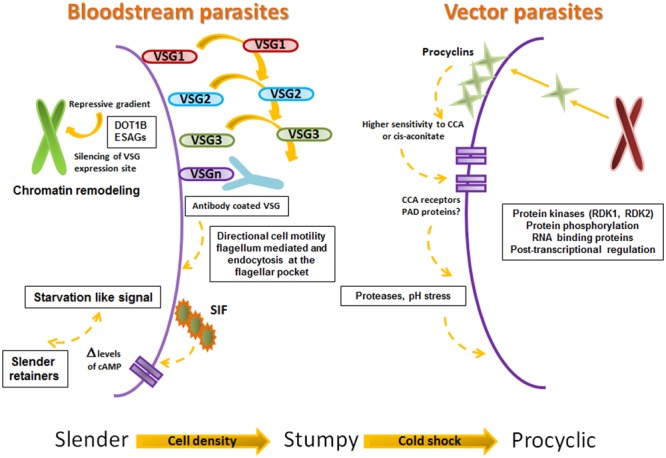
**Proposed steps on Slender, Stumpy, Procyclic progression.** Framework for the successive progression of trypanosomes from slender to stumpy to procyclic stages. The figure summarizes the data proposed by Engstler and Boshart (2004), Engstler et al. (2007), Figueiredo et al. (2008), Hertz-Fowler et al. (2008), Field and Carrington (2009), Horn (2014), and Mony and Matthews (2015).

**FIGURE 4:**
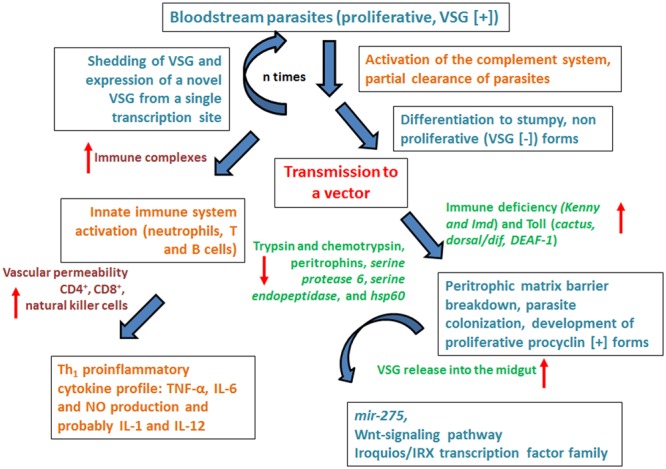
**Overview of the lively host–parasite-vector interaction in *Trypanosoma brucei* infections.** The figure summarizes the dynamics of host–parasite interaction emphasizing the role of the immune system both of the human host and the vector. For details please see main text.

What happens at the cellular level, which mechanisms and which molecules are involved in the host–parasite interaction? The answer is so far focused on the VSG (see **Figures 3** and **4**). The VSG is abundantly expressed on the membrane of parasites belonging to the genus *Trypanosoma*, being the predominant surface antigen of African trypanosomes. It is highly immunogenic, and one of the main components of the molecular interface that mediates the host–parasite interaction. Exposure of the VSG coat at the surface of trypanosomes, rather than being a tool to avoid the immune system, challenges it to produce a lytic antibody response (Pays et al., 2001). In fact, *via* the complement system, VSG-specific antibodies facilitate efficient opsonization and lysis of parasites expressing the coat against which the response was triggered (**Figure 4**). Trypanosomes avoid detection by host antibodies by recurrently switching to new VSG coats (Horn and Mcculloch, 2010); i.e., once antibody titer increase, the vast majority of parasites are eliminated and only cells with distinct VSG coats survive (**Figure 3**, Horn, 2014).

Antigenic variation of *T. brucei* was originally described in Franke (1905). However, an understanding of the evolution and expression (or not) of VSG by each parasite, the variety of the expressed VSG, as well as the range of sequence similarities among the VSGs expressed by strains and isolates is far from complete (Cross et al., 2014). Current information regarding VSGs relies on data obtained from the TREU927 strain (Cross et al., 2014), and the partial catalog of VSG genes is described in: http://tritrypdb.org/common/downloads/release-5.0/, http://www.genedb.org/Homepage/Tbruceibrucei927

A repertoire of hundreds of VSG genes with a single active transcription site, always at the end of a chromosome immediately adjacent to the telomere, has been described in the parasite genome (Pays et al., 2001; Horn and Mcculloch, 2010). The new VSG to be expressed and become active must be copied into the single transcribed locus by DNA repair processes. Additionally, a novel VSG gene can be activated by the so called allelic exclusion mechanism. This is a coordinated transcriptional switch that does not produce detectable changes in the DNA sequence, and maintains singular expression (Horn, 2014). This permits a continuous stochastic switching of VSG genes at high rates per cell generation, depending on strain and parasite growing conditions (Turner and Barry, 1989; Turner, 1997). Incomplete genes or pseudogenes, which exist as single dynamic and highly mutated copies with a strong codon-usage bias, predominate (ca 80%) on the VSG-genome (Cross et al., 2014).

The VSG role as a protective “cell coat” is activated in the tsetse fly salivary gland (before infecting the human host), functions during the bloodstream mammalian infectious cycle and is inactivated upon return to the tsetse fly midgut. Bloodstream parasites express only one VSG coat at a time. Under the pressure to express a different VSG, a repressive gradient extends from the chromosome end, shutting down the active VSG expression site (Horn and Mcculloch, 2010). The silencing of this expression site triggers trypanosomes to move on from the proliferative (bloodstream-stage) to the non-proliferative form, as a pre-adaptation for life in the insect (**Figures 3** and **4**).

It is not completely clear how silencing is produced. Chromatin remodeling triggers the silencing to initiate the change of trypanosome performance from proliferative to non-proliferative. The success of this event depends on the function of a DOT histone methyltransferase (DOT1B; Figueiredo et al., 2008) together with attenuation of the expression produced at some VSG ESAGs (Hertz-Fowler et al., 2008). Furthermore, it has been proposed that some ESAGs could function as ‘sensors’ that cue trypanosomes to become quiescent when the expression site shuts down (**Figures 3** and **4**, Batram et al., 2014).

The VSG coat is “fluid” and permits a high rate recycling. VSG-switch and coat exchange starts by dilution during cell division, with cells dividing approximately every 6 h and shedding and turnover being relatively much slower (Seyfang et al., 1990). In the meantime, the coat is cleansed of antibodies, aided by the vigorous directional cell motility mediated by the flagellum (Engstler et al., 2007; Horn, 2014) and endocytosis at the flagellar pocket (Field and Carrington, 2009; Horn, 2014). In conclusion, the role of VSG at the human host–parasite interface is extremely active. The reiteration of VSG function (expression-shedding) trigger complex responses that in the end might signify the exhaustion of the immune system, the survival of the parasite and the transmission to the vector (**Figures 3** and **4**).

### Vector Parasites

Stumpy forms only differentiate efficiently to procyclic forms within the tsetse midgut (Vickerman, 1985; Matthews and Gull, 1994), despite their exposure to the acidic pH environment and the action of proteases (Nolan et al., 2000). In the tsetse fly procyclic forms replace (in a coupled process) the VSG coat by procyclins, molecules that do not protect the parasite from lysis by serum components; this means that if differentiation to the procyclic form occurs at the wrong time and place, i.e., within the mammalian host, the parasites would definitively die (**Figures 3** and **4**, Wenzler et al., 2016).

High concentrations (>3 mM, higher than expected in the tsetse midgut) of citrate or CCA induce *in vitro* the differentiation of bloodstream form trypanosomes to procyclic forms (Brun and Schonenberger, 1981; Hunt et al., 1994). Also, a drop in temperature from 37 to 20°C, similar to that encountered by trypanosomes when sucked into the tsetse flies’ midgut, induces the reversible expression of procyclins on the surface of trypanosomes and increases (stumpy form) trypanosome sensitivity to CCA (down to μM concentrations, Engstler and Boshart, 2004). This suggests that CCA levels present within the tsetse fly blood-meal could be physiologically relevant as the parasite reaches the digestive tract of the tsetse fly at dawn or sunset when temperatures are mild.

Based on these data, Engstler and Boshart (2004) proposed a hierarchical model with three steps that sequentially regulate differentiation once transmission to the vector (or drop in temperature) occur (**Figure 3**). They base their model on the fact that the order of events strictly depends on the “out of homeotherm” information provided by the cold shock. This initial (first step) triggering signal (or step) promotes the function of a surface system that permits “routing” of the procyclins to the surface (only in stumpy form parasites and transiently coexisting with VSG). The next steps need the increased (>1000-fold) sensitivity of parasites to the differentiation chemical signal (CCA or citrate), and a concomitant expression of surface receptors for this signal (Engstler and Boshart, 2004). Although all the data is not yet available to confirm the role of CCA and citrate in parasite differentiation, Engstler and Boshart (2004) suggest that CCA and citrate could be mimics of a yet-unidentified compound or activation mechanism present in the vector.

The nature of the molecule responsible for the transmission of the CCA differentiation signal has thus been elusive. However, a gene family encoding surface carboxylate transporters known as PAD proteins (proteins associated with differentiation, PAD1 and PAD2), expressed in stumpy forms but not in slender forms, share properties that make them good candidates to perform this function. PAD proteins are expressed on the surface of stumpy-form parasites present in the bloodstream. At least PAD2 is thermoregulated, its expression is confined to the parasite’s flagellar pocket region at 37°C and spreads to the cell surface at 20°C; finally, inhibition of PAD expression by the use of RNAi diminishes CCA-induced differentiation and eliminates CCA-sensitivity under cold-shock conditions (Brun and Schonenberger, 1981; Engstler and Boshart, 2004; Dean et al., 2009). This suggests that PAD proteins may act in coordination with the CCA signal system, providing stringent control of differentiation (**Figure 3**).

Exposure to proteases or pH stress in the tsetse midgut might also participate in triggering differentiation, either in a complementary or a parallel manner (Rolin et al., 1998; Sbicego et al., 1999). Newly enclosed tsetse flies (teneral) have an immature immune system and are less prone to be infected by trypanosomes, when compared with mature adults (Aksoy et al., 2016). In experimentally infected mature tsetse flies, genes related with DNA/RNA binding, protein phosphorylation, and initiation of transcription, as well as with cytoskeleton and actin binding cell-remodeling functions are upregulated. Signaling pathway (immune deficiency) genes related with the immune system (*Kenny and Imd*) and Toll (*cactus, dorsal/dif, DEAF-1)* are also upregulated. On the other hand, major gut enzyme transcripts (trypsins and chymotrypsins) and those related to the peritrophic matrix barrier formation (peritrophins, *serine protease 6, serine endopeptidase*, and *hsp60*) are down regulated. These facts suggest that levels of effector molecules present in the gut at parasite arrival may interfere with parasite colonization or survival. Additionally, they suggest that trypanosome-mediated inhibition of peritrophic matrix functions might reduce the action of the vector immune system and facilitate the translocation of the parasite into the ectoperitrophic space where parasites reside in infected flies (**Figures 3** and **4**, Aksoy et al., 2016).

Additionally, the release of the parasite VSG into the midgut of the tsetse fly supports successful colonization of the gut. Free VSG induces a decreased expression of a micro RNA (*mir-275*). In *Aedes aegypti mir-275* is linked to gut function and blood digestion processes (Bryant et al., 2010). It also interferes with the Wnt-signaling pathway and the Iroquois/IRX transcription factor family (Cavodeassi et al., 2001; Lucas et al., 2015). The Wnt-signaling pathway mediates in eukaryotes cell proliferation, cell-to-cell communication and embryonic development; in the adult mosquito, it is involved in fat body secretory processes (Logan and Nusse, 2004). The Iroquois/IRX family regulates embryonic and larval development mechanisms in *Drosophila*. The integrated action of these processes compromises midgut homeostasis and the integrity of the semipermeable, chitinous barrier that lines the peritrophic matrix (Aksoy et al., 2016), thus illustrating how active the vector-parasite interaction is (**Figures 3** and **4**).

## Parasite-Human Host Interaction

The following sections deal with the tools that the parasite uses to evade the consequences of the human immune system activation, and that the human host uses to promote the successful clearance of the parasite.

### The Role of Parasite and Host Genetics

The genetic variation of the parasite influences the outcome of the disease. This depends on the specific parasite involved in causing it. In fact, pathogenesis is strain specific, at least partially, and may involve different host mechanisms. For example, *T. b. rhodesiense* causes the acute HAT disease and *T. b. gambiense*, the more chronic infection (Barrett et al., 2003). Most information obtained up to date related to the role of parasite genetics in the outcome of the disease has been described in experimental infections made in animals; the results suggest that the expression of trypano-tolerance or the existence of trypano-tolerant hosts (mammals that remain infected but do not display the full-blown disease) are the consequence of the performance of specific genetic loci.

In fact, trypano-tolerance can be understood as the reduction of disease pathogenic consequences. The genetic loci (called QTL) involved in this phenomenon have been described in cattle and in experimental models of infected mice (Morrison et al., 1978; Murray and Morrison, 1979; Murray et al., 1981, 1982; Kemp et al., 1997; Hill et al., 2015). QTLs identified in cattle do not overlap completely with those described in mice, and are much larger; however, both share IL-10 and TNF-α genes as related to mice and cattle susceptibility. These facts have been useful to understand genetic and immunological background of infection produced by trypanosome species in non-primate mammals, but still extrapolation to what occurs in human beings is not yet straight forward.

In fact, in human, the definition of trypano-tolerance has been questioned, although reports on asymptomatic carriers and spontaneous cure have been published (Bucheton et al., 2011). For example, in HAT caused by the *gambiense* strain (considered as invariably fatal), cases of self-cure in untreated patients have been described, although accurate diagnostic tools were not available at the time (revised by Jamonneau et al., 2012). In studies performed in mangrove areas of coastal Guinea, high levels of IL-10 and low levels of TNF-α have been associated with an increased risk to suffer HAT, whereas high levels of IL-8 were associated with disease control (Ilboudo et al., 2014). Recent data from patients that refused to receive chemotherapy and were monitored for periods from 5 to 15 years demonstrated parasite clearance, as observed using follow-up tools such as microscopy and polymerase chain reaction. Most of the patients became progressively negative to trypanosome variable antigens, and expressed milder serological responses (Jamonneau et al., 2012). This means that whether trypano-tolerance in humans is related to intrinsic host or parasite factors, or relies on genetic grounds or on heavy host-T-cell immunity, or on parasite virulence factors, or on a combination of these, remains to be determined.

Finally, it is well known that clinical outcomes of “mild” and “severe” *T. b. rhodesiense* HAT show a correlation with the geographical area from which patients come (Hide and Tait, 1991). This is so for example for Malawi and Uganda (MacLean et al., 2004), but also for restricted geographical areas of Uganda (MacLean et al., 2007). Whether or not this relies on a trypano-tolerant phenotype in which the host immunology is determinant must be further analyzed (Morrison et al., 2010).

### Parasite Tools to Modulate the Host Response in Human African Trypanosomiasis

#### Quorum Sensing

Replication of slender forms present in the blood of infected hosts increases the parasitemia. This does not occur *ad infinitum*; trypanosomes control their levels within mammalian hosts. An equilibrium exists –at any time during the infection- as cell proliferation should be enough to guarantee infection transmission without surpassing the balance that would destroy the host, at least at early phases of disease. The initial infecting parasites may be cleared by the immune system; but as, we have previously learned, a small proportion of the trypanosome population switches coats and these parasites with a different antigenic specificity at their surface escape from the immune system and remain in the bloodstream long enough to allow a successful transmission to new hosts (**Figures 3** and **4**, Vincendeau and Bouteille, 2006; Horn and Mcculloch, 2010; Morrison, 2011; Horn, 2014).

Cells, including trypanosomes, are able to sense their environment and respond accordingly. In fact, differentiation of slender to quiescent, stumpy bloodstream forms is an individual event that occurs independently of the host, even in the absence of an immune response (Seed and Sechelski, 1988; Vassella et al., 1997). So, even *in vitro*, pleomorphic strains of trypanosomes undergo differentiation into stumpy forms once a cell density is reached. These facts suggest the existence of a density signal that would help to arrest growth of parasites to hamper their further proliferation (Hesse et al., 1995; Reuner et al., 1997; Vassella et al., 1997), thus working like a quorum sensing mechanism. This parasite–parasite signaling system, as well as the fact that at peak parasitemia the plasma from infected animals inhibits trypanosome proliferation, suggests that a secreted factor could work like the molecule that controls parasite switch from proliferation to differentiation (**Figures 3** and **4**, Mony and Matthews, 2015).

Trypanosomes (pleomorphs and monomorphic) secrete a soluble, low molecular weight, heat stable factor/s termed SIF. This is a molecule proposed to be segregated by slender form parasites that accumulates in the culture medium/bloodstream, upon increasing parasite density. It is proposed to function as a slender to stumpy trigger molecule that acts through autocrine mechanisms (Vassella et al., 1997). Although the identity and mechanism of action of SIF are still not completely elucidated, its effector systems seem to include cAMP or hydrolysis products of cAMP (Vassella et al., 1997; Laxman et al., 2006); however, controversial recent results using the chemical inducer (8-cPT-cAMP/AMP) ruled out the use of the canonical pathway of cAMP (Laxman et al., 2006). Although most of the data needs to be validated, it suggests that several protein families are implicated in the control of stumpy formation (Mony and Matthews, 2015). This includes networks that either work to retain the slender form of the parasite or promote stumpy form differentiation mimicking a starvation like signal. This signaling further activates protein kinases that induce G1/G0 arrest; protein phosphatases that regulate protein kinase activities, inactivate slender retainers and deactivate inhibitors of stumpy differentiation; reactivates mitochondrial metabolism and leads to the regulation of genes needed for stumpy form maintenance and differentiation to procyclic forms (**Figures 3** and **4**, Mony and Matthews, 2015). A detailed description is beyond the scope of this review but this mechanism has been thoroughly analyzed previously (Mony and Matthews, 2015).

#### Antigenic Diversity

As already mentioned, due to the mechanism of antigenic variation the expression of a novel VSG constitutes the main way of evading a constant attack by the immune system. Thus, individual cells switch the identity of the expressed VSG at a low frequency, to be selected (or not) by the host immune response. If the VSG is novel, it acts as a protective barrier by shielding the cell from innate and adaptive immune factors, and trypanosomes proliferate, thus maintaining the infection; this happens until an overwhelming titer of antibodies recognize the expressed VSG (Pays, 2005). If VSG doesn’t switch, or if the new VSG is not novel, the parasite will be killed. Additionally, antibodies produced by the host are eliminated from the parasite surface by the concerted action of clathrin-dependent endocytosis of antibody-bound surface proteins and antibody degradation that aids immune evasion while the host antibody titer is low (O’Beirne et al., 1998; Hall et al., 2003; Manna et al., 2013).

### Host Tools to Tackle the Infection in Human African Trypanosomiasis

#### Innate Immunity

The interaction between trypanosomes and their mammalian hosts triggers events that sequentially activate innate and specific immunity. The efficient presentation of parasitic antigens leads to the activation of T and B cells and the development of strictly regulated effector mechanisms. However, since trypanosomes have efficiently “learned” to cope with the host immune system, the effectivity of the triggered responses is limited and the parasites are seldom completely eliminated thus inducing immunopathological phenomena that end up in tissue damage (**Figure 4**, Vincendeau and Bouteille, 2006).

In HAT, when trypanosome enters the skin of the mammalian host upon the tsetse fly bite, trypanosome proliferation induces a local skin reaction that constitutes the first protection to be developed by the host. This skin reaction eventually transforms in an ulcer (the chancre). The lymphatic vessels surrounding it already have dividing trypanosomes ca. 5 days after the bite and a couple of days before the complete establishment of the chancre. Eventually the response is mainly mediated by T cells (specially TCD8^+^, see **Figure 4**, Vincendeau and Bouteille, 2006).

Although complement activation is commonly detected at this initial stage of HAT, it is also usual to observe “hypocomplementemia” (Devine et al., 1986). This has been attributed to an activation (and eventual depletion) of the complement pathway directly by the parasites, as has been demonstrated in *T*. *b. gambiense* exposed to human serum. The complement activation occurs without triggering a lytic activity, the reason being that the cascade does not continue beyond the establishment of C3 convertase on the trypanosome surface (Devine et al., 1986). Also, a time dependent decrease in complement activity has been observed during trypanosome infections by *T. b. gambiense* or *T. b. rhodesiense* (Matthews et al., 2015).

This all means that the initial immune response is complex. In fact, parasite clearance may occur via antibody-mediated lysis due to the activation of the classical complement pathway (through specific antibodies against trypanosomes), or via the alternative complement pathway, independent of specific antibodies, (on procyclic, VSG [-] trypanosomes). Of note, VSG isolated from *T. b. brucei* activates the classical complement pathway in an antibody-independent fashion [**Figure 4**, (Musoke and Barbet, 1977)]. On the other hand, immune complexes constituted by antibodies specific to trypanosomes (e.g., anti- VSG antibodies) may arise (Russo et al., 1994; Vincendeau and Bouteille, 2006). These immune complexes, together with complement pathway activation, promote tissue damage that might induce aggressive effects including thrombosis and renal glomerular damage (Hasselbalch et al., 1985; Bruijn et al., 1988; van Velthuysen et al., 1994).

The activation of the complement pathway may lead to the appearance of soluble fragments. These could include the C3a and C5a anaphylatoxins, and the complex C567. Some of these fragments may trigger chemotactic responses by neutrophils and monocytes, as well as the release of neurotransmitters involved in triggering an increase on vascular permeability that aids the initial inflammatory response to the chancre. Finally, natural killer cells might also participate in the initiation of the inflammatory response through the synthesis of cytokines and chemokines like IFN-γ and TNF-α (**Figure 4**, Vincendeau and Bouteille, 2006).

Information about the role of cell mediated immune responses is mainly described in animal models of experimental infections. The results suggest that neutrophils and T and B lymphocytes infiltrate and predominate in the local skin reaction, the former in the early days whereas the latter permeate the chancre at 5–7 days post-infection, with CD8^+^ T cells predominating in the chancre at even later stages (Mwangi et al., 1990; Vincendeau and Bouteille, 2006) and increased density of CD4^+^ and CD8^+^ T cells in the afferent lymph draining the chancre. In this context, VSG causes a polyclonal B-cell activation that triggers the generation of auto-antibodies (Kazyumba et al., 1986) and immune complexes (Lambert et al., 1981). The healing of the chancre occurs simultaneously with an increase in lymphoblast and surface immunoglobulin bearing cells in the afferent draining lymph, while in the efferent lymph there is an increase in lymphocytes bearing surface immunoglobulins (Mwangi et al., 1996; Vincendeau and Bouteille, 2006). The effectiveness of the immune system at this stage is limited since parasites cannot be eliminated due to their genetic versatility (Barry and Emergy, 1984). Then, differentiation to stumpy forms occur (Vincendeau and Bouteille, 2006).

#### Cytokines and Chemokines

Trypanosomes are prone to activate the innate immune system early in infection, a mechanism that affects B- and T-cell responses to parasite antigens, including VSG as well as many other surface and intracellular antigens like cell-free DNA. For example, the activation of macrophages, as well as of additional antigen presenting cells, are dramatically altered in trypanosome infected tissues leading to a highly polarized Th_1_ proinflammatory cytokine profile that includes TNF-α, IL-6 and NO production and probably IL-1 and IL-12 (**Figure 4**, reviewed by Mansfield and Paulnock, 2005).

The first evidence of the dysregulation of the cytokine network that occurs in HAT is the induction by VSG of TNF-α (over) production by macrophages (Rouzer and Cerami, 1980; Tachado and Schofield, 1994; Okomo-Assoumou et al., 1995; Magez et al., 2002). High levels of TNF-α and patent inflammatory signs then occur in the early phase of human trypanosomiasis, as well as during major neurologic manifestations in the late phase (Okomo-Assoumou et al., 1995). This cytokine plays a role on activation, proliferation and differentiation of B cells (Rouzer and Cerami, 1980; Roldán et al., 1992), triggering a cascade of events leading to elimination of parasites (Lucas et al., 1993). At the clinical level, TNF-α induces fever, asthenia, cachexia, and hypertriglyceridemia (Vincendeau and Bouteille, 2006); additionally, increased serum TNF-α levels contribute to the hypergammaglobulinemia observed in trypanosomiasis.

On the other hand, interferon-gamma (IFN-γ) has a key role in relative resistance to African trypanosomes. This cytokine is transiently released primarily by parasite antigen activated Th_1_ cells, together with natural killer cells at earlier time points in infection, activated by the TLTF released by the parasites. Additionally, TGF-β, which has immunosuppressive effects, is secreted by CD8 T cells also activated by the TLTF released by trypanosomes (Olsson et al., 1991; Vaidya et al., 1997; reviewed by Mansfield and Paulnock, 2005).

All in all, the parasite is capable of interfering with the cytokine network, and can use cytokines as growth factors. By doing that, trypanosomes modify the effector functions of the immune system (Hide et al., 2004). Moreover, in the CNS of *T. b. brucei*-infected animals, chemokines favor macrophage and lymphocyte recruitment to areas where their activity might induce additional alterations (Buguet et al., 1993, 2001; Lundkvist et al., 2004). These results together with the fact that TNF-α RNA transcripts have been described in the CNS and that intracerebral infusion of soluble type I TNF-α receptor reduced trypanosome-induced neurodegeneration (Quan et al., 2003), suggest that TNF-α production could play a role in CNS disorders and that TNF-α and other cytokines might contribute to the generation of somnogenic molecules such as IL-1 (Pentreath et al., 1994).

## Conclusion

Antigenic variation is one of several resources by which trypanosomes manipulate their hosts. VSG of trypanosomes constitutes the main antigenic molecule exposed in the surface of these blood parasites. The mechanisms by which antigen switching occurs are sophisticated and are likely to contribute to infection chronicity. Coat renewal by VSG-switch is a strategy by which the parasite successfully eludes the immune system, avoids the action of blood-borne antibodies, and continues to successfully infect the human host. Therefore, the effectiveness of the immune system is limited and the parasite remains in the bloodstream long enough to permit transmission to a new host, either blood-sucking insects or human *via* blood–blood contact. Superimposed upon antigen switching, the density-dependent production of cell-cycle arrested parasite transmission stages limits the infection and ensures parasite spread to new hosts via the bite of blood feeding tsetse flies, thus contributing to trypanosome infection transmission. Neither antigen switching nor developmental progression to transmission stages is driven by the host. However, the human host contributes to the infection dynamics through the selection of distinct antigen types, his/her genetic susceptibility (trypanotolerance) and the potential influence of host-dependent effects on parasite pathogenicity. Although further activation of innate immunity and cytokine and chemokine secretion foster the successful clearance of the parasite by the human host, the interaction between the human host and the parasite is extremely active and leads to responses that need multiple control sites to develop appropriately. The complexity of the involved events reveals that trypanosomes use a focused strategy that questions the existence of a truly developed human host response.

## Author Contributions

The author confirms being the sole contributor of this work and approved it for publication.

## Conflict of Interest Statement

The author declares that the research was conducted in the absence of any commercial or financial relationships that could be construed as a potential conflict of interest.

## Abbreviations

CCA: *cis*-aconitate
CNS: central nervous system
CSF: cerebrospinal fluid
DOT: disruptor-of telomeric silencing
ESAGs: expression sites associated genes
HAT: human African trypanosomiasis
PAD: protein associated with differentiation
SIF: stumpy induction factor
TLF: trypanolytic factors
TLTF: trypanosome triggering factor
VSG: variant surface glycoprotein
